# Supplementary material for: Hybrid Models and Biological Model Reduction with PyDSTool
Source: PLoS Comput Biol. 2012 Aug 9;8(8):e1002628. doi: 10.1371/journal.pcbi.1002628 (PMC3415397; doi:10.1371/journal.pcbi.1002628)
Supplement: Text S4 — Complete source code for the PyDSTool package (version 0.88.120504). Includes API documentation and help files linking to web pages. This file is identical to the current public release on Sourceforge.net. (ZIP) [file pcbi.1002628.s004.zip › PyDSTool/html/PyDSTool.Toolbox.optimizers.step-module.html]

xml version="1.0" encoding="ascii"?


PyDSTool.Toolbox.optimizers.step


| Home | Trees | Indices | Help | PyDSTool |
| --- | --- | --- | --- | --- |

Package PyDSTool ::
Package Toolbox ::
Package optimizers ::
Package step

[hide private]

[frames] | no frames]

# Package step

source code

Module containing every step use to lower a cost function

Steps :

- GradientStep
  - compute a step based on the gradient of the function
- CWConjugateGradient
  - Crowder-Wolfe conjugate gradient
- DYConjugateGradient
  - Dai-Yuan conjugate gradient
- DConjugateGradient
  - Dixon conjugate gradient
- FRConjugateGradient
  - Fletcher-Reeves conjugate gradient
- PRPConjugateGradient
  - Polak-Ribiere-Polyak conjugate gradient
- FRPRPConjugateGradient
  - Fletcher-Reeves modified Polak-Ribiere-Polyak conjugate gradient
- NewtonStep
  - computes a step based on the hessian and the gradient of the
    function
- MarquardtStep
  - computes a step based on the Marquardt modified hessian and the
    gradient of the function
- GoldsteinPriceStep
  - computes a step based on the Goldstein-Price Newton modification
- GoldfeldStep
  - computes a step based on the Goldfeld Newton modification
- DFPNewtonStep
  - computes a step based on the Davidson-Fletcher-Powell
    approximation of the hessian
- PartialStep
  - decorator for another step but uses only part of this step
- RestartPeriodicallyConjugateGradientStep
  - decorator for a conjugate gradient step that restarts the
    conjugation each n iterations
- RestartNotOrthogonalConjugateGradientStep
  - decorator for a conjugate gradient step that restarts the
    conjugation if the last gradients are not orthogonal enough
- LocalBruteForce1Dstep
  - local brute force step in 1D (experimental)


|  |  |
| --- | --- |
| Submodules | [hide private] |

- **PyDSTool.Toolbox.optimizers.step.conjugate\_gradient\_step**: *Computes the conjugate gradient steps for a specific function at a
  specific point*
- **PyDSTool.Toolbox.optimizers.step.goldfeld\_step**: *Computes Goldfeld step for a specific function at a specific point*
- **PyDSTool.Toolbox.optimizers.step.goldstein\_price\_step**: *Computes Goldstein-Price step for a specific function at a specific
  point*
- **PyDSTool.Toolbox.optimizers.step.gradient\_step**: *Computes a gradient step for a specific function at a specific
  point*
- **PyDSTool.Toolbox.optimizers.step.local\_brute\_force\_1dstep**
- **PyDSTool.Toolbox.optimizers.step.marquardt\_step**: *Computes a Marquardt step for a specific function at a specific
  point*
- **PyDSTool.Toolbox.optimizers.step.newton\_step**: *Computes a Newton step for a specific function at a specific point*
- **PyDSTool.Toolbox.optimizers.step.partial\_step**: *Computes a partial step for a specific function at a specific
  point, acting like a decorator for other steps*
- **PyDSTool.Toolbox.optimizers.step.quasi\_newton\_step**: *Computes a quasi-Newton step for a specific function at a specific
  point*
- **PyDSTool.Toolbox.optimizers.step.restart\_conjugate\_gradient**: *Restarts a conjugate gradient search by deleting the step key in
  the state dictionary*

  


|  |  |
| --- | --- |
| Classes | [hide private] |

GradientStep  
The simple gradient step

NewtonStep  
The Newton step

MarquardtStep  
The simple gradient step

GoldfeldStep  
The Goldfeld step

GoldsteinPriceStep  
The Goldstein-Price step

PartialStep  
A partial step

RestartPeriodicallyConjugateGradientStep  
A step decorator that periodically deletes the direction key in the
dictionary so that the CG search can be restarted

RestartNotOrthogonalConjugateGradientStep  
A step decorator that deletes the direction key in the dictionary
if the last gradients are not orthogonal enough so that the CG
search can be restarted

LocalBruteForce1DStep  
Local brute force search for 1D parameter (sub-)space, making no
use of gradient information.


|  |  |
| --- | --- |
| Functions | [hide private] |

|  |  |
| --- | --- |
| DYConjugateGradientStep()  The Dai Yan conjugate gradient step Has good convergence capabilities (same as the FR-PRP gradient) | source code |

|  |  |
| --- | --- |
| FRPRPConjugateGradientStep()  The Fletcher-Reeves modified Polak-Ribiere-Polyak conjugate gradient step Can restart automatically and has the advantages of the PRP gradient and of the FR gradient | source code |

|  |  |
| --- | --- |
| PRPConjugateGradientStep()  The Polak-Ribiere-Polyak conjugate gradient step Can restart automatically, but needs an exact line search with a uniformely convex function to globally converge | source code |

|  |  |
| --- | --- |
| CWConjugateGradientStep()  The Crowder-Wolfe or Hestenes-Stiefel conjugate gradient step | source code |

|  |  |
| --- | --- |
| FRConjugateGradientStep()  The Fletcher Reeves conjugate gradient step Needs an exact line search for convergence or the strong Wolfe-Powell rules for an inexact line search | source code |

|  |  |
| --- | --- |
| DConjugateGradientStep()  The Dixon conjugate gradient step | source code |


|  |  |
| --- | --- |
| Variables | [hide private] |

step\_\_all\_\_ = `['GradientStep', 'CWConjugateGradientStep', 'DYC...`


|  |  |
| --- | --- |
| Variables Details | [hide private] |

### step\_\_all\_\_

Value:
:   ```
    ['GradientStep',
     'CWConjugateGradientStep',
     'DYConjugateGradientStep',
     'DConjugateGradientStep',
     'FRConjugateGradientStep',
     'PRPConjugateGradientStep',
     'FRPRPConjugateGradientStep',
     'NewtonStep',
    ...
    ```

  


| Home | Trees | Indices | Help | PyDSTool |
| --- | --- | --- | --- | --- |

|  |  |
| --- | --- |
| Generated by Epydoc 3.0.1 on Fri May 4 15:24:05 2012 | http://epydoc.sourceforge.net |
